# Supplementary material for: Structure and immunogenicity of a stabilized HIV-1 envelope trimer based on a group-M consensus sequence
Source: Nat Commun. 2019 May 29;10:2355. doi: 10.1038/s41467-019-10262-5 (PMC6541627; doi:10.1038/s41467-019-10262-5)
Supplement: Supplementary file 1 — Supplementary Information [file 41467_2019_10262_MOESM1_ESM.pdf]

Structure and immunogenicity of a stabilized HIV-1 envelope trimer based on a group-M consensus sequence

Sliepen, Han, Bontjer et al.

```
                                                                      49
                TPA leader                                          47 49           64
HxB2 gp160      MRVKEKYQHLWRWGWRWGTMLLGMLMICS------ATEKLWVTVYYGVPVWKEATTTLFCASDAKAYDTE
ConM gp160      MRVMGIQRNCQHL-WRWGILIFGMLIICS------AAENLWVTVYYGVPVWKEANTTLFCASDAKAYDTE
ConS gp160      MRVRGIQRNCQHL-WRWGTLILGMLMICS------AAENLWVTVYYGVPVWKEANTTLFCASDAKAYDTE
ConM SOSIP.v7   MDAMKRGLCCVLL-LCGAVFVSPSQEIHARFRRGARAENLWVTVYYGVPVWKDAETTLFCASDAKAYDTE
BG505 SOSIP.664 MDAMKRGLCCVLL-LCGAVFVSPSQEIHARFRRGARAENLWVTVYYGVPVWKDAETTLFCASDAKAYETK
BG505 SOSIP.v6  MDAMKRGLCCVLL-LCGAVFVSPSQEIHARFRRGARAENLWVTVYYGVPVWKDACTTLFCASDAKAYETK

               6566   73                              106
HxB2 gp160      VHNVWATHACVPTDPNPQEVVLVNVTENFNMWKNDMVEQMHEDIISLWDQSLKPCVKLTPLCVSLKCTDL
ConM gp160      VHNVWATHACVPTDPNPQEIVLENVTENFNMWKNNMVEQMHEDIISLWDQSLKPCVKLTPLCVTLNCTDV
ConS gp160      VHNVWATHACVPTDPNPQEIVLENVTENFNMWKNNMVEQMHEDIISLWDQSLKPCVKLTPLCVTLNCTNV
ConM SOSIP.v7   KRNVWATHCCVPTDPNPQEIVLENVTENFNMWKNNMVEQMHTDIISLWDQSLKPCVKLTPLCVTLNCTDV
BG505 SOSIP.664 KHNVWATHACVPTDPNPQEIHLENVTEEFNMWKNNMVEQMHTDIISLWDQSLKPCVKLTPLCVTLQCTNV
BG505 SOSIP.v6  KHNVWATHCCVPTDPNPQEIHLENVTEEFNMWKNNMVEQMHTDIISLWDQSLKPCVKLTPLCVTLQCTNV

                                                  165
HxB2 gp160      KNDTNTNSSSGR-----MIMEKGEIKNCSFNISTSIRGKVQKEYAFFYKLDIIPIDNDTTS--------Y
ConM gp160      NATNNTTNNEE-------------IKNCSFNITTEIRDKKKKVYALFYKLDVVPIDDNNS---------Y
ConS gp160      NVTNTTNNTEEK----------GEIKNCSFNITTEIRDKKQKVYALFYRLDVVPIDDNNNNSSN-----Y
ConM SOSIP.v7   NATNNTTNNEE-------------IKNCSFNITTELRDKKKKVYALFYKLDVVPIDDNNS---------Y
BG505 SOSIP.664 T-NNITDDMR------------GELKNCSFNMTTELRDKKQKVYSLFYRLDVVQINENQGNRSNNSNKEY
BG505 SOSIP.v6  T-NNITDDMR------------GELKNCSFNMTTELRDKKQKVYSLFYRLDVVQINENQGNRSNNSNKEY

HxB2 gp160      KLTSCNTSVITQACPKVSFEPIPIHYCAPAGFAILKCNNKTFNGTGPCTNVSTVQCTHGIRPVVSTQLLL
ConM gp160      RLINCNTSAITQACPKVSFEPIPIHYCAPAGFAILKCNDKKFNGTGPCKNVSTVQCTHGIKPVVSTQLLL
ConS gp160      RLINCNTSAITQACPKVSFEPIPIHYCAPAGFAILKCNDKKFNGTGPCKNVSTVQCTHGIKPVVSTQLLL
ConM SOSIP.v7   RLINCNTSAITQACPKVSFEPIPIHYCAPAGFAILKCNDKKFNGTGPCKNVSTVQCTHGIKPVVSTQLLL
BG505 SOSIP.664 RLINCNTSAITQACPKVSFEPIPIHYCAPAGFAILKCKDKKFNGTGPCPSVSTVQCTHGIKPVVSTQLLL
BG505 SOSIP.v6  RLINCNTSAITQACPKVSFEPIPIHYCAPAGFAILKCKDKKFNGTGPCPSVSTVQCTHGIKPVVSTQLLL

                                                                     316
HxB2 gp160      NGSLAEEEVVIRSVNFTDNAKTIIVQLNTSVEINCTRPNNNTRKRIRIQRGPGRAFVTIG-KIGNMRQAH
ConM gp160      NGSLAEEEIIIRSENITNNAKTIIVQLNESVEINCTRPNNNTRKSIRI--GPGQAFYATGDIIGDIRQAH
ConS gp160      NGSLAEEEIIIRSENITNNAKTIIVQLNESVEINCTRPNNNTRKSIRI--GPGQAFYATGDIIGDIRQAH
ConM SOSIP.v7   NGSLAEEEIIIRSENITNNAKTIIVQLNESVEINCTRPNNNTRKSIRI--GPGQWFYATGDIIGDIRQAH
BG505 SOSIP.664 NGSLAEEEVMIRSENITNNAKNILVQFNTPVQINCTRPNNNTRKSIRI--GPGQAFYATGDIIGDIRQAH
BG505 SOSIP.v6  NGSLAEEEVMIRSENITNNAKNILVQFNTPVQINCTRPNNNTRKSIRI--GPGQWFYATGDIIGDIRQAH

HxB2 gp160      CNISRAKWNNTLKQIASKLREQFGNNKTIIFKQSSGGDPEIVTHSFNCGGEFFYCNSTQLFNSTWFNSTW
ConM gp160      CNISRTKWNKTLQQVAKKLREHFN-KTII-FNPSSGGDLEITTHSFNCGGEFFYCNTSELFNSTWNG---
ConS gp160      CNISGTKWNKTLQQVAKKLREHFNNKTII-FKPSSGGDLEITTHSFNCRGEFFYCNTSGLFNSTWIGN--
ConM SOSIP.v7   CNISRTKWNKTLQQVAKKLREHFN-KTII-FNPSSGGDLEITTHSFNCGGEFFYCNTSELFNSTWNG---
BG505 SOSIP.664 CNVSKATWNETLGKVVKQLRKHFGNNTIIRFANSSGGDLEVTTHSFNCGGEFFYCNTSGLFNSTWISN-T
BG505 SOSIP.v6  CNVSKATWNETLGKVVKQLRKHFGNNTIIRFANSSGGDLEVTTHSFNCGGEFFYCNTSGLFNSTWISN-T

                                           429 432
HxB2 gp160      STEGSNNTEGSDTITLPCRIKQIINMWQKVGKAMYAPPISGQIRCSSNITGLLLTRDGGNSNNE-SEIFR
ConM gp160      ---------TNNTITLPCRIKQIINMWQGVGQAMYAPPIEGKIRCTSNITGLLLTRDGGNNNT---ETFR
ConS gp160      --GTKNNNNTNDTITLPCRIKQIINMWQGVGQAMYAPPIEGKITCKSNITGLLLTRDGGNNNTNETEIFR
ConM SOSIP.v7   ---------TNNTITLPCRIKQIINMWQRVGQAMYAPPIEGKIRCTSNITGLLLTRDGGNNNT---ETFR
BG505 SOSIP.664 SVQGSNSTGSNDSITLPCRIKQIINMWQRIGQAMYAPPIQGVIRCVSNITGLILTRDGGSTNST-TETFR
BG505 SOSIP.v6  SVQGSNSTGSNDSITLPCRIKQIINMWQRIGQAMYAPPIQGVIRCVSNITGLILTRDGGSTNST-TETFR

                                            500501                                535
HxB2 gp160      PGGGDMRDNWRSELYKYKVVKIEPLGVAPTKAKRRVVQREKR--AVGIGALFLGFLGAAGSTMGAASMTL
ConM gp160      PGGGDMRDNWRSELYKYKVVKIEPLGVAPTKAKRRVVEREKR--AVGIGAVFLGFLGAAGSTMGAASITL
ConS gp160      PGGGDMRDNWRSELYKYKVVKIEPLGVAPTKAKRRVVEREKR--AVGIGAVFLGFLGAAGSTMGAASITL
ConM SOSIP.v7   PGGGDMRDNWRSELYKYKVVKIEPLGVAPTRCKRRVVERRRRRRAVGIGAVFLGFLGAAGSTMGAASMTL
BG505 SOSIP.v6  PGGGDMRDNWRSELYKYKVVKIEPLGVAPTRCKRRVVGRRRRRRAVGIGAVFLGFLGAAGSTMGAASMTL
BG505 SOSIP.v6  PGGGDMRDNWRSELYKYKVVKIEPLGVAPTRCKRRVVGRRRRRRAVGIGAVFLGFLGAAGSTMGAASMTL

                    543        555 559 561                                            605
HxB2 gp160      TVQARQLLSGIVQQQNNLLRAIEAQQHLLQLTVWGIKQLQARILAVERYLKDQQLLGIWGCSGKLICTTA
ConM gp160      TVQARQLLSGIVQQQSNLLRAIEAQQHLLQLTVWGIKQLQARVLAVERYLKDQQLLGIWGCSGKLICTTN
ConS gp160      TVQARQLLSGIVQQQSNLLRAIEAQQHLLQLTVWGIKQLQARVLAVERYLKDQQLLGIWGCSGKLICTTT
ConM SOSIP.v7   TVQARNLLSGIVQQQSNLLRAPECQQHLLQLTVWGIKQLQARVLAVERYLKDQQLLGIWGCSGKLICCTN
BG505 SOSIP.664 TVQARNLLSGIVQQQSNLLRAPEAQQHLLKLTVWGIKQLQARVLAVERYLRDQQLLGIWGCSGKLICCTN
BG505 SOSIP.v6  TVQARNLLSGIVQQQSNCLRAPECQQHLLKLTVWGIKQLQARVLAVERYLRDQQLLGIWGCSGKLICCTN

                                                                       664
HxB2 gp160      VPWNASWSNKSLEQIWNHTTWMEWDREINNYTSLIHSLIEESQNQQEKNEQELLELDKWASLWNWFNITN
ConM gp160      VPWNSSWSNKSQDEIWDNMTWMEWDKEINNYTDIIYSLIEESQNQQEKNEQELLALDKWASLWNWFDITN
ConS gp160      VPWNSSWSNKSQDEIWDNMTWMEWEREINNYTDIIYSLIEESQNQQEKNEQELLALDKWASLWNWFDITN
ConM SOSIP.v7   VPWNSSWSNKSQDEIWDNMTWMEWDKEINNYTDIIYSLIEESQNQQEKNEQELLALD-------------
BG505 SOSIP.v6  VPWNSSWSNRNLSEIWDNMTWLQWDKEISNYTQIIYGLLEESQNQQEKNEQDLLALD-------------
BG505 SOSIP.v6  VPWNSSWSNRNLSEIWDNMTWLQWDKEISNYTQIIYGLLEESQNQQEKNEQDLLALD-------------
```

Supplementary Figure 1. Amino acid sequence alignment of ConM SOSIP.v7 with HxB2 gp160, ConM gp160 (alignment ID: 104CP2), ConS gp160, BG505 SOSIP.664 and BG505 SOSIP.v6. The SOSIP design, trimer-stabilizing and improved trimerization mutations are indicated in bold[1]; the extra intermolecular disulfide bond in red[2]; and the TD8 mutations in blue[3] (HxB2 numbering).

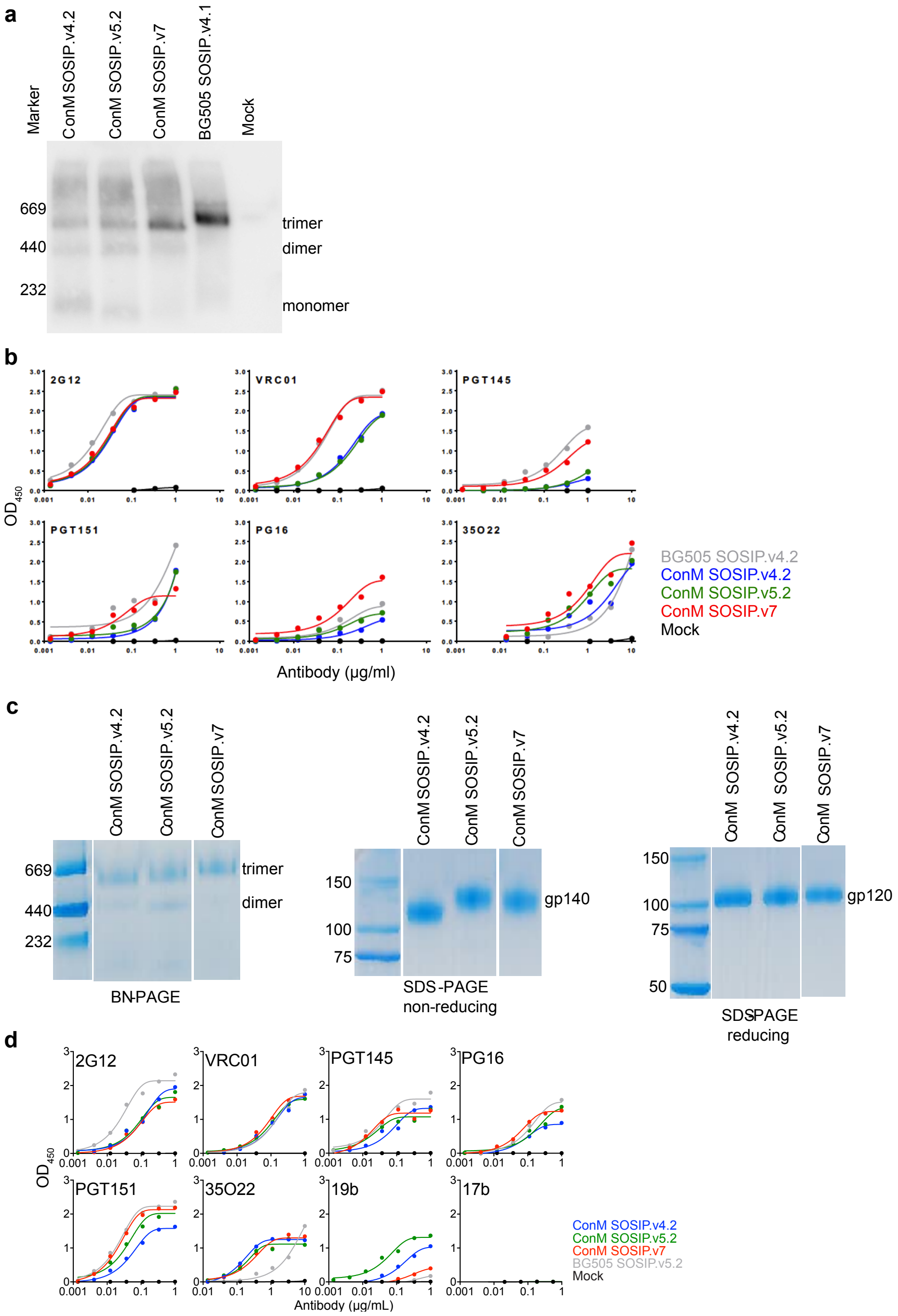

Supplementary Figure 2. Screening and characterization of ConM SOSIP.v4.2, SOSIP.v5.2 and SOSIP.v7 trimers. **a** BN-PAGE analysis of unpurified D7324-tagged SOSIP trimers expressed in 293T cells followed by Western blotting with 2G12/VRC01. **b** D7324-capture ELISA with unpurified D7324-tagged SOSIP trimers. **c** PGT145-purified SOSIP trimers were analyzed by BN-PAGE analysis, reducing (+DTT) and non-reducing (-DTT) SDS-PAGE followed by Coomassie Blue staining. We note the difference in migration pattern of SOSIP.v4 and SOSIP.v5 trimers in non-reducing SDS-PAGE gels, which are consistent with previous results showing that SOSIP.v5 proteins, which contain an extra intraprotomer disulfide bond between the gp120 and gp41, migrate more slowly than their SOSIP.664 and SOSIP.v4 counterparts. The likely explanation is a decrease in SDS uptake when a protein becomes more compact [2]. **d** D7324-capture ELISA with PGT145-purified ConM SOSIP trimer (D7324-tagged) expressed in 293F cells against a panel of bNAbs.

a

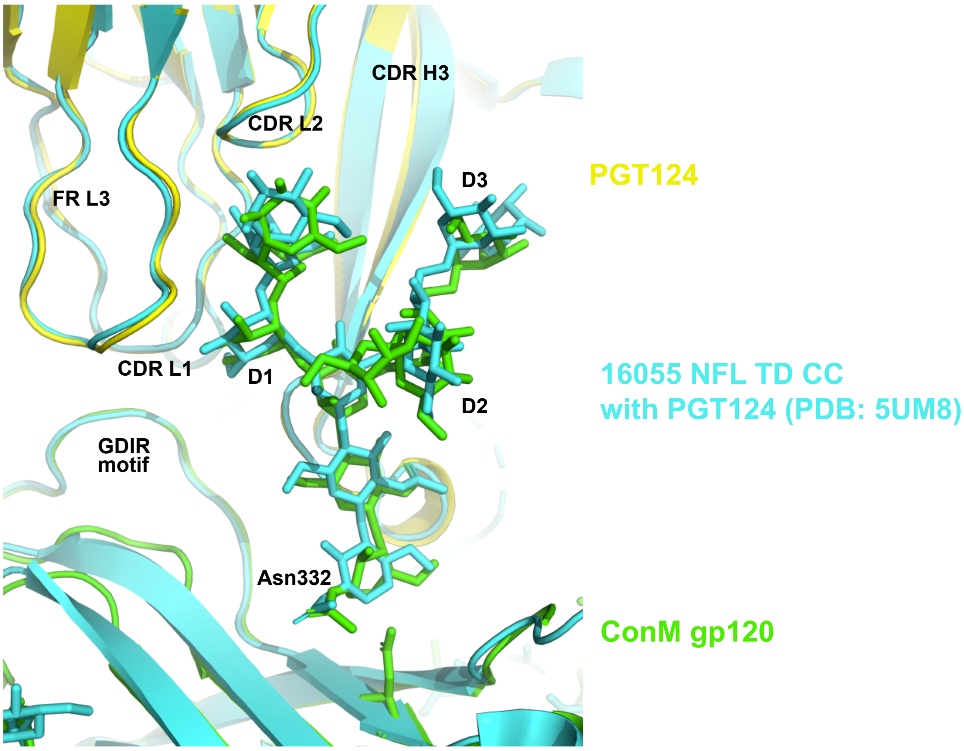

b

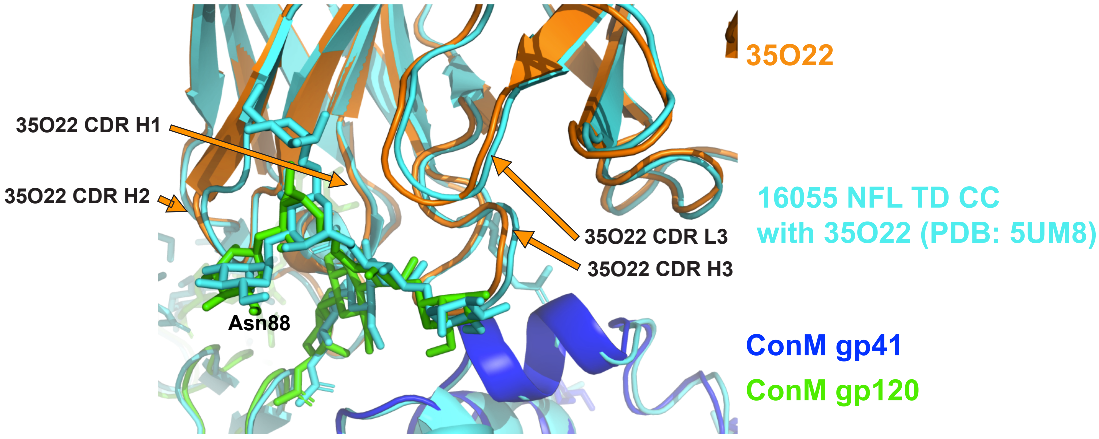

Supplementary Figure 3. Binding modes of PGT124 and 35O22 to ConM SOSIP.v7. **a.** Interaction of the CDR loops of PGT124 in yellow with the oligomannose glycan at the Asn332 supersite in green stick representation and ^{324}GDIR^{327} motif at the V3 loop base. The ^{324}GDIR^{327} motif and D1/D2/D3 arms in the oligomannose glycan at Asn332 are labeled. The structure of 16055 NFL TD CC in complex with PGT124 and 35O22 is represented in cyan cartoon for comparison. **b.** Interaction of the CDR loops of 35O22 in orange with the gp120/gp41 interface. gp120 and gp41 are represented as green and blue cartoon, respectively.

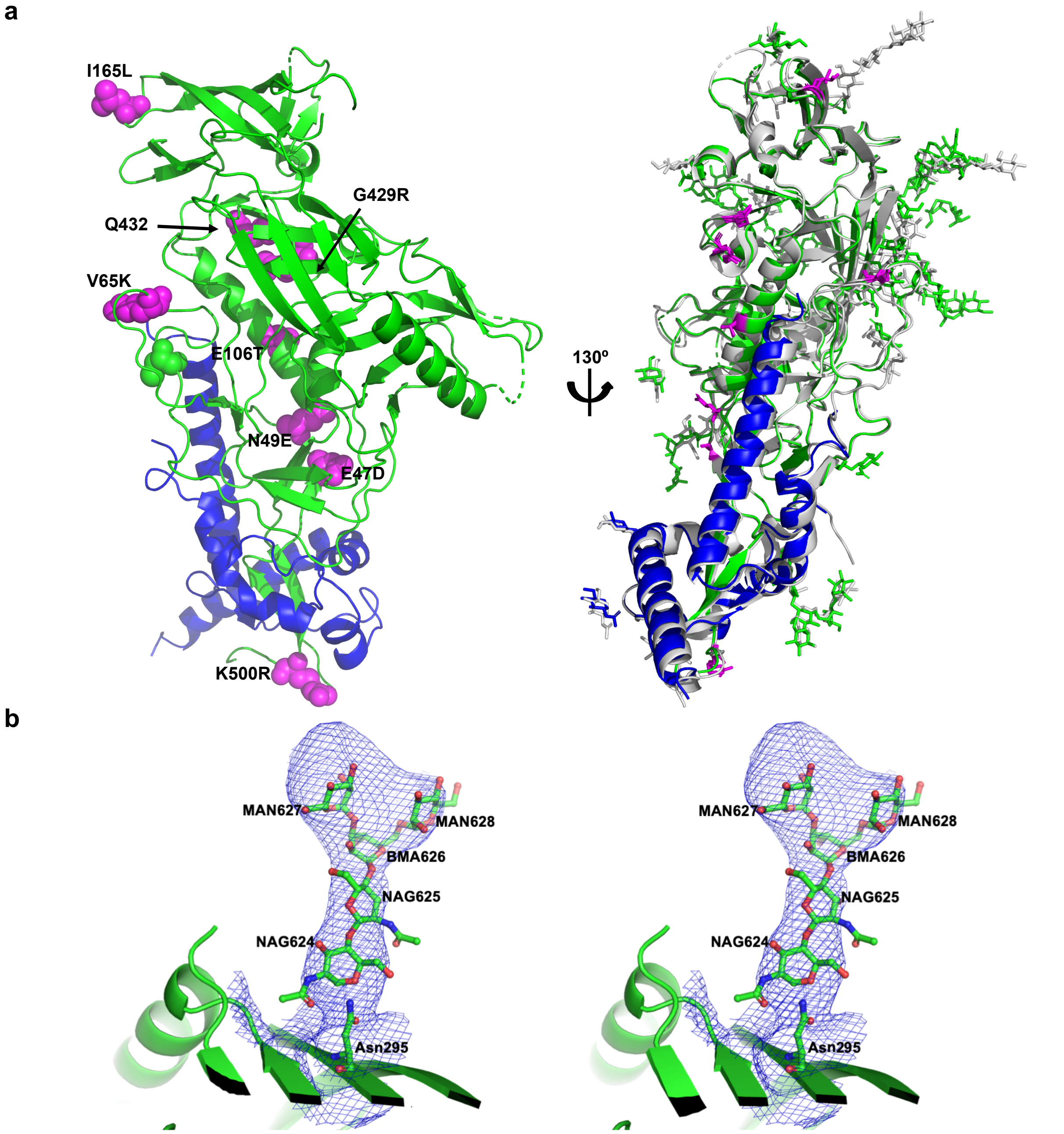

Supplementary Figure 4. Structural features of ConM SOSIP.v7. **a.** Characteristics of TD8 residues of ConM SOSIP.v7. All TD8 residues (E47D, N49E, V65K, E106T, I165L, G429R, Q432, and K500R) could be resolved in the crystal structure of ConM SOSIP.v7 in complex with bNAbs PGT124 and 35O22. Gp120 and gp41 are represented by green and blue cartoons, respectively, and TD8 residues are shown as magenta spheres (left panel). Superposition of ConM SOSIP.v7 with BG505 SOSIP.664 (PDB ID: 5CEZ) shows that the TD8 residues in ConM SOSIP.v7 are structurally similar to those in BG505 SOSIP.664 in stabilizing the Env trimers (right panel). The BG505 SOSIP.664 structure is represented in gray cartoon model and the TD8 residues in ConM SOSIP.v7 and BG505 SOSIP.664 are shown in magenta stick models. **b.** Stereo image of Asn295 and linked glycans (NAG624-MAN628). 2m*Fo*-D*Fc* electron density maps are shown at 1.0σ. Asn295 and linked glycans are represented by stick models, and carbon, nitrogen, and oxygen atoms are shown in green, blue, and red, respectively.

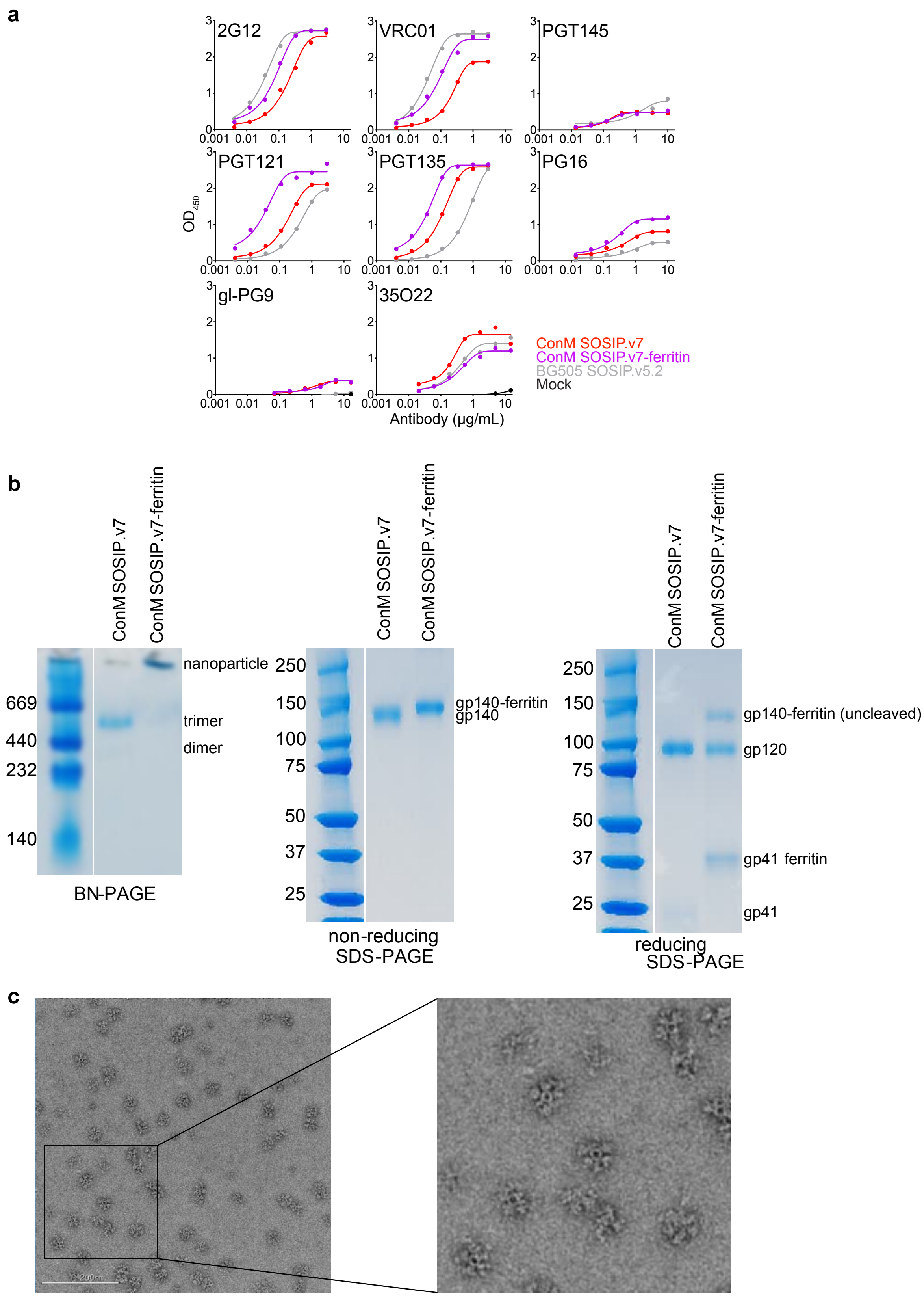

Supplementary Figure 5 Characterization of ConM SOSIP.v7-ferritin nanoparticles. **a.** Lectin-capture ELISA with PGT145-purified ConM SOSIP.v7 trimers and ConM SOSIP.v7-ferritin nanoparticles expressed in 293F cells. **b.** PGT145-purified SOSIP-ferritin was analyzed by BN-PAGE analysis, reducing (+DTT) and non-reducing (-DTT) SDS-PAGE followed by Coomassie Blue staining. **c.** NS-EM images of PGT145-purified ConM SOSIP.v7-ferritin.

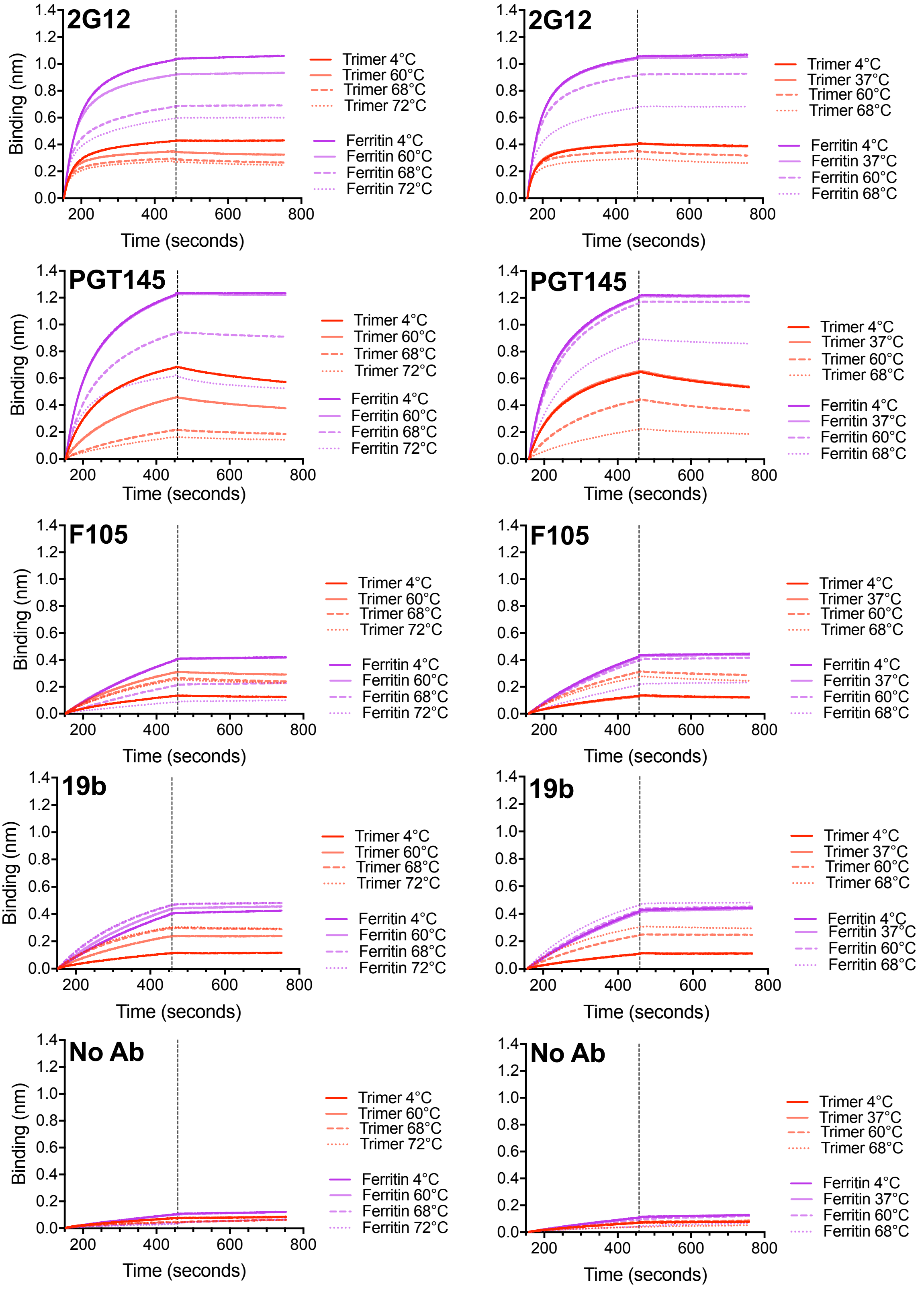

Supplementary Figure 6. Thermostability of antibody epitopes on ConM SOSIP.v7 trimers and nanoparticles. BLI binding curves of 2G12, PGT145, F105 and 19b to ConM SOSIP.v7 trimers and ConM SOSIP.v7-ferritin nanoparticles incubated at different temperatures. The results of two independent experiments are shown (left and right panels) and the average AUC values are plotted in Fig. 5f.

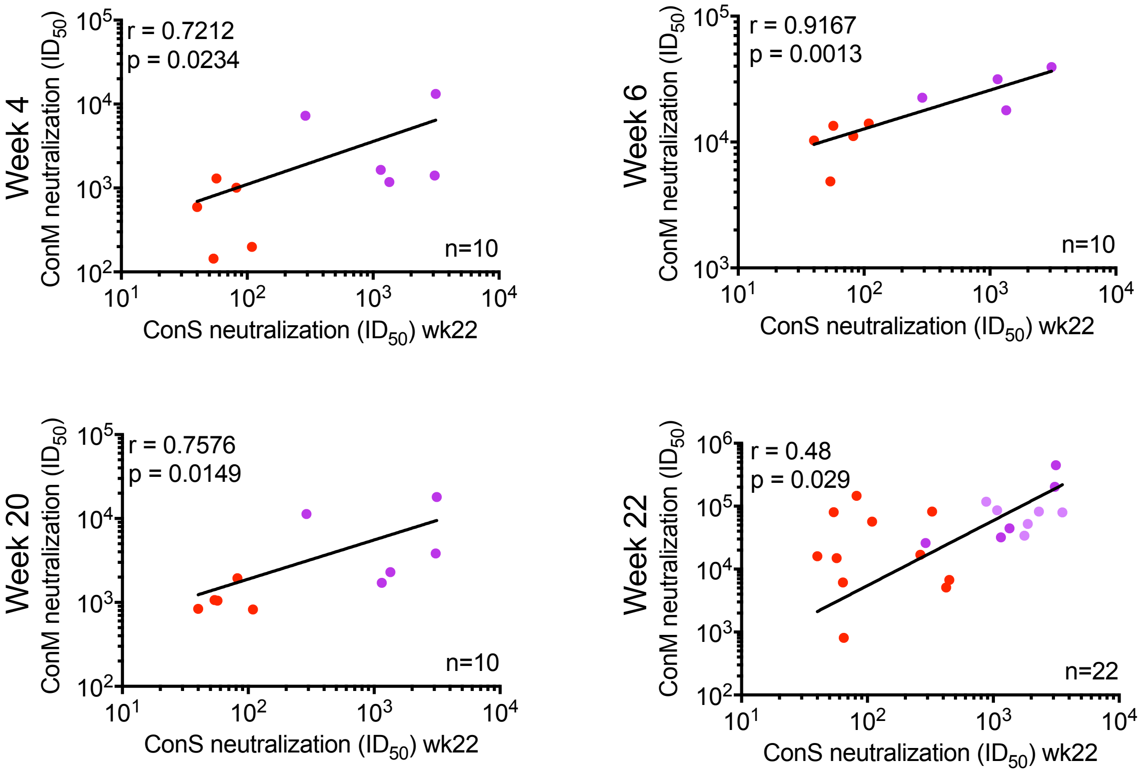

Supplementary Figure 7. Correlation between ConM and ConS neutralization. Plots show the correlation between the neutralization titers (ID_{50}) of the autologous Tier 1A virus at different timepoints with the related Tier 1B ConS virus at week 22. Titers against ConM for weeks 4, 6 and 20 are from Fig. 4C (n=10) and for week 22 from Supplementary Table 4 (n=22). ConM trimer immunized animals are in red and the nanoparticle immunized animals in magenta. Spearman r and p values are given.

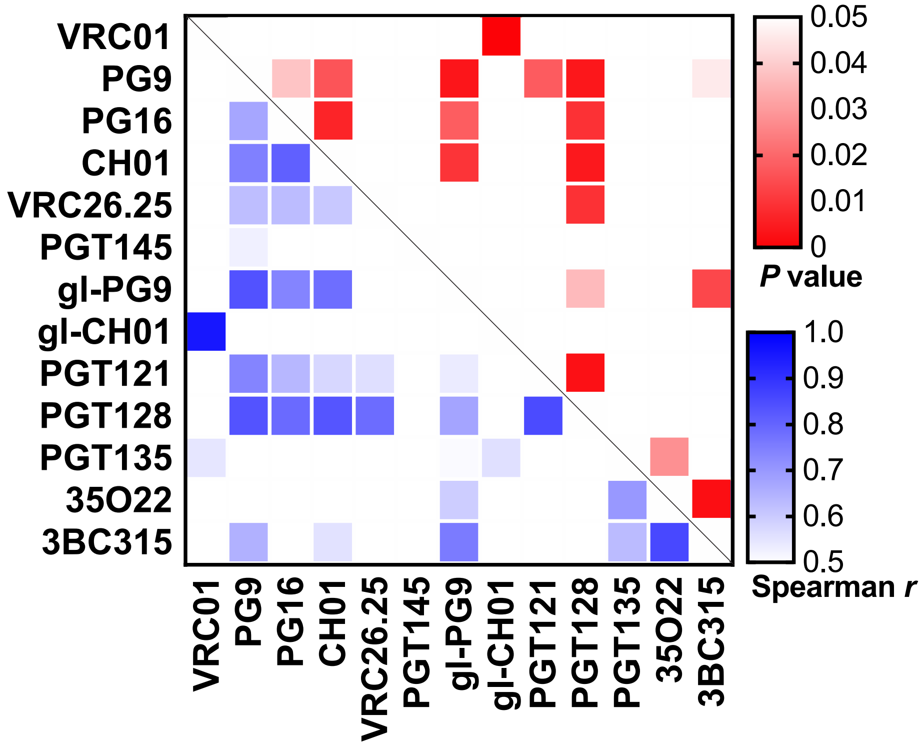

Supplementary Figure 8. Correlation matrix of bNAb binding interference by sera of ConM immunized rabbits. The competition ELISA values are shown in Fig. 5a. Spearman r values are represented in blue of increasing intensity and the corresponding P values are in red of increasing intensity.

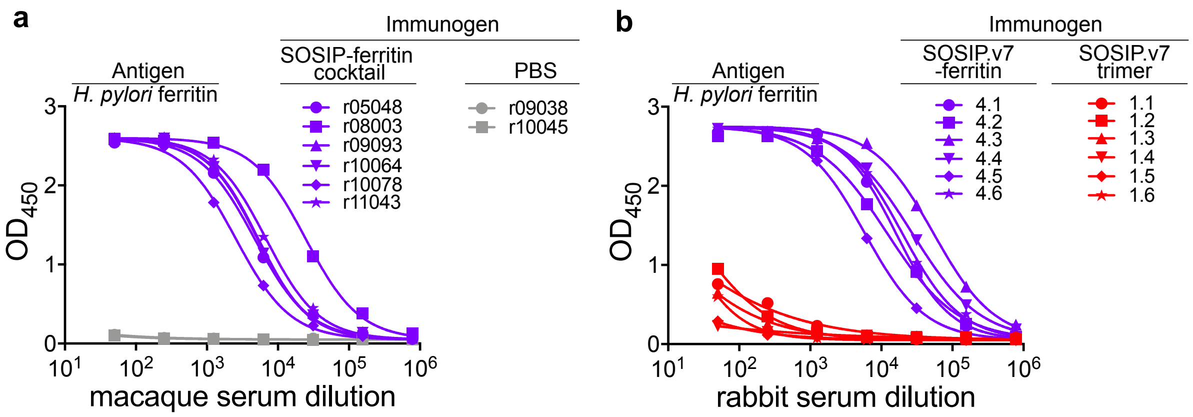

Supplementary Figure 9. Immunogenicity of the ferritin component of ConM SOSIP.v7 ferritin nanoparticles. ELISA binding curves against His-tagged ferritin (devoid of Env) using week 22 sera from immunized macaques (**a**) and rabbits (**b**).

			501C-605C	I559P	R6	ΔMPER	64K	66R	316W	535M	543Q	543N	72C-564C	73C-561C	49C-555C	47D [c]	49E [c]	65K [c]	106T [c]	165L [c]	429R [c]	432Q [c]	500R [c]
SOSIP.681	SOSIP.v1	SOSIP.v1	green	green	green	red	red	red	red	red	red	red	red	red	red	red	red	red	red	red	red	red	red
SOSIP.664	SOSIP.v2	SOSIP.v2	green	green	green	green	red	red	red	red	red	red	red	red	red	red	red	red	red	red	red	red	red
	SOSIP.v3	SOSIP.v3.1	green	green	green	green	red	red	red	green	green	red	red	red	red	red	red	red	red	red	red	red	red
		SOSIP.v3.2	green	green	green	green	red	red	red	green	red	green	red	red	red	red	red	red	red	red	red	red	red
	SOSIP.v4 [a]	SOSIP.v4.1	green	green	green	green	green	red	green	green	green	green	red	red	red	red	red	red	red	red	red	red	red
		SOSIP.v4.2	green	green	green	green	red	green	green	green	green	green	red	red	red	red	red	red	red	red	red	red	red
	SOSIP.v5 [b]	SOSIP.v5.1	green	green	green	green	green	green	green	green	green	green	green	red	red	red	red	red	red	red	red	red	red
		SOSIP.v5.2	green	green	green	green	green	green	green	green	green	green	red	green	red	red	red	red	red	red	red	red	red
	SOSIP.v6 [b]	SOSIP.v6	green	green	green	green	green	green	green	green	green	green	red	green	green	red	red	red	red	red	red	red	red
	SOSIP.v7	SOSIP.v7	green	green	green	green	green	green	green	green	green	green	red	green	red	green	green	green	green	green	green	green	green

Supplementary Table 1. Nomenclature for the stabilized ConM SOSIP trimers, related to Fig. 1b. The modifications included in each SOSIP version are indicated in green (present) and red (not present).

[a] described in de Taeye et al. 2015[1]

[b] described in Torrents de la Peña et al. 2017[2]

[c] described in Guenaga et al. 2015[3]

	ConM SOSIP.v7		Clade A (BG505, PDB 5FYL)		Clade B (JR-FL, PDB 5FYK)		Clade G (X1193.c1, PDB 5FYJ)	
N glycan	Residue	Glycan moieties (residue number in PDB file)	N glycan	Glycan moieties	N glycan	Glycan moieties	N glycan	Glycan moieties
N88	N88	6 (601-606)	N88	7	N88	7	N88	8
N130	N130	1 (607)						
N133	D133		N133	2			N133	1
N135	N135				N135	1		
N137	T137		N137	7				
N142	N142						N142	1
N156	N156		N156	9	N156	9	N156	11
N160	N160	1 (608)	N160	8	N160	7	N160	9
N187	N187				N187	7		
N188	N188						N188	10
N190								
N190C								
N197	N197	1 (609)	N197	6			N197	3
N234	N234	4 (610-613)	N234	5			N234	6
N241	N241	2 (614-615)			N241	4	N241	7
N262	N262	7 (616-622)	N262	8	N262	9	N262	11
N276	N276	1 (623)	N276	5	N276	8	N276	9
N293	E293						N293	9
N295	N295	5 (624-628)	N295	7	N295	5		
N301	N301	2 (629-630)	N301	8	N301	8	N301	6
N332	N332	10 (631-639, 635A)	N332	10	N332	10	N332	10
N339	N339		N339	1	N339	8		
N344	Q344						N344	7
N355	N355		N355	1	N355	7	N355	3
N362	N362				N362	4		
N363	P363		N363	3				
N386	N386	5 (640-644)	N386	6	N386	8	N386	8
N392	N392	2 (645-646)	N392	6	N392	6	N392	1
N397	N396				N397	2		
N413	N411 or N412						N413	10
N442	K442						N442	6
N448	N448	1 (647)	N448	6	N448	6		
N462	N462							
N463	N463				N463	2		
N464	N464						N464A	2
N611	N611	1 (701)	N611	1	N611	3	N611	2
N616	N616				N616	1	N616	2
N618	S618		N618	1				
N625	N625				N625	1	N625	3
N637	N637	1 (702)	N637	3	N637	1	N637	2

Supplementary Table 2. Glycans observed in the ConM SOSIP.v7 structure in complex with PGT124 and 35O22. Possible Asn sequons were summarized from BG505 SOSIP.664 (clade A, [4]), BG505 (clade A, PDB: 5FYL), JR-FL (clade B, PDB: 5FYK), and X11.93.c1 (clade G, PDB: 5FYJ) [5].

Residue No.	ConM gp140 wt	Amino acid conservation rate	BG505 gp140 T332N wt	Amino acid conservation rate	Conservation rate gap between ConM and BG505	Consensus
630	E	35.23%	Q	62.40%	27.17%	Q 62.40%; E 35.23%; D 1.37%; K 0.54%; A 0.15%; X 0.10%; R 0.07%; G 0.05%; L 0.02%; M 0.02%; P 0.02%; S 0.02%
636	N	17.49%	S	40.61%	23.12%	S 40.61%; D 32.72%; N 17.49%; E 3.88%; G 2.63%; I 0.83%; H 0.51%; A 0.41%; R 0.28%; T 0.23%; X 0.11%; Q 0.10%; F 0.05%; Y 0.05%; K 0.03%; V 0.03%; C 0.02%
152	E	22.15%	G	41.51%	19.36%	G 41.51%; E 22.15%; D 10.47%; K 3.93%; T 3.70%; N 3.58%; R 2.39%; S 2.23%; Q 2.08%; I 1.96%; A 1.60%; V 1.16%; M 0.79%; P 0.61%; X 0.56%; L 0.52%; Y 0.11%; W 0.08%; H 0.07%; C 0.05%; F 0.02%
646	I	40.72%	L	58.97%	18.25%	L 58.97%; I 40.72%; X 0.08%; F 0.05%; T 0.03%; A 0.02%; C 0.02%; H 0.02%; P 0.02%; R 0.02%; S 0.02%; V 0.02%; Y 0.02%
412	N	4.97%	D	23.09%	18.12%	D 23.09%; G 17.24%; S 14.69%; E 13.60%; T 10.47%; N 4.97%; K 4.27%; A 3.58%; R 1.52%; I 1.36%; L 0.92%; V 0.90%; P 0.80%; Q 0.61%; X 0.57%; Y 0.21%; H 0.18%; F 0.11%; M 0.10%; C 0.05%; W 0.02%
170	K	30.76%	Q	46.88%	16.12%	Q 46.88%; K 30.76%; R 9.55%; H 2.13%; E 2.11%; T 1.44%; L 1.01%; N 0.69%; S 0.46%; M 0.26%; X 0.20%; V 0.16%; I 0.13%; Y 0.13%; A 0.07%; D 0.07%; F 0.03%; P 0.03%; W 0.03%; # 0.02%; G 0.02%
389	E	4.52%	G	18.91%	14.39%	Q 24.97%; K 24.53%; G 18.91%; N 6.09%; R 5.97%; E 4.52%; P 3.81%; S 3.37%; D 2.63%; A 1.88%; I 0.80%; L 0.70%; T 0.67%; H 0.38%; X 0.29%; V 0.16%; Y 0.15%; C 0.03%; # 0.02%; F 0.02%; M 0.02%; W 0.02%
619	Q	21.32%	L	34.96%	13.64%	L 34.96%; Y 21.91%; Q 21.32%; F 3.81%; E 3.66%; H 3.19%; K 2.77%; V 2.34%; M 2.21%; I 1.28%; R 1.05%; W 0.33%; X 0.26%; T 0.21%; S 0.18%; D 0.13%; N 0.13%; A 0.08%; G 0.03%; * 0.02%; P 0.02%
133	D	36.45%	N	43.98%	7.53%	N 43.98%; D 36.45%; E 5.50%; K 3.62%; T 2.59%; S 2.05%; H 1.52%; A 1.16%; Y 0.70%; Q 0.46%; X 0.39%; P 0.34%; G 0.33%; I 0.29%; R 0.16%; M 0.10%; V 0.10%; C 0.05%; L 0.05%; F 0.03%; * 0.02%
410	T	16.03%	S	21.19%	5.16%	S 21.19%; T 16.03%; N 15.13%; G 11.52%; E 6.77%; D 6.66%; L 3.03%; A 2.86%; C 2.32%; K 2.18%; I 2.01%; P 1.95%; R 1.47%; V 1.37%; Q 0.75%; Y 0.70%; H 0.46%; X 0.38%; F 0.34%; M 0.29%; W 0.10%; # 0.03%
446	T	8.88%	V	11.53%	2.65%	S 31.81%; K 12.30%; V 11.53%; N 10.52%; T 8.88%; E 7.00%; R 6.22%; I 4.61%; L 3.30%; A 1.19%; D 1.16%; Q 0.28%; H 0.25%; X 0.20%; M 0.18%; P 0.15%; G 0.13%; F 0.08%; Y 0.07%; C 0.05%; # 0.02%; * 0.02%
151	N	4.89%	R	7.53%	2.64%	K 19.78%; T 15.85%; E 10.44%; G 7.80%; R 7.53%; N 4.89%; D 4.22%; S 3.71%; I 3.57%; Q 3.45%; P 3.34%; V 2.83%; A 2.65%; M 2.23%; Y 1.98%; L 1.01%; X 0.93%; C 0.49%; W 0.49%; F 0.34%; H 0.29%; * 0.05%
442	K	5.53%	V	7.84%	2.31%	N 29.14%; Q 21.34%; I 7.95%; V 7.84%; L 6.59%; K 5.53%; E 3.93%; S 3.88%; P 3.78%; R 3.53%; T 1.98%; Y 1.00%; A 0.98%; H 0.82%; D 0.67%; M 0.54%; X 0.13%; F 0.11%; W 0.11%; G 0.07%; C 0.03%; # 0.02%
186	D	16.34%	E	16.43%	0.09%	N 35.00%; E 16.43%; D 16.34%; G 12.42%; K 7.82%; S 6.23%; T 1.29%; R 1.05%; A 0.80%; Q 0.62%; P 0.61%; X 0.31%; H 0.25%; V 0.25%; I 0.23%; L 0.08%; M 0.07%; Y 0.07%; C 0.02%; F 0.02%
397	G	11.78%	S	11.75%	-0.03%	N 12.91%; G 11.78%; S 11.75%; E 4.65%; P 4.63%; D 4.45%; T 4.32%; A 3.71%; F 3.53%; V 3.24%; K 3.22%; W 2.65%; I 2.55%; R 2.42%; L 1.75%; Y 1.18%; H 1.08%; Q 0.75%; X 0.74%; M 0.43%; C 0.08%; * 0.02%
137	T	33.56%	N	31.51%	-2.05%	T 33.56%; N 31.51%; S 7.98%; A 4.22%; K 3.22%; I 3.17%; D 3.08%; V 2.95%; G 2.01%; E 1.16%; R 1.15%; P 0.88%; X 0.75%; L 0.72%; H 0.64%; Y 0.51%; C 0.47%; Q 0.29%; F 0.23%; M 0.11%; W 0.08%; # 0.05%
189	S	4.27%	G	0.95%	-3.32%	N 6.99%; S 4.27%; K 1.47%; T 1.37%; D 1.15%; G 0.95%; E 0.69%; I 0.51%; A 0.34%; P 0.34%; R 0.34%; V 0.28%; Q 0.23%; H 0.18%; Y 0.10%; M 0.05%; L 0.03%; X 0.03%
440	E	12.70%	Q	7.72%	-4.98%	A 27.80%; R 20.21%; S 14.76%; E 12.70%; K 10.00%; Q 7.72%; P 4.40%; N 0.52%; G 0.47%; T 0.44%; D 0.29%; X 0.20%; I 0.13%; M 0.13%; V 0.10%; L 0.07%; H 0.03%; C 0.02%
346	A	32.98%	V	24.71%	-8.27%	A 32.98%; V 24.71%; S 11.01%; K 9.44%; G 7.64%; T 6.00%; R 4.66%; E 1.18%; I 0.80%; Q 0.34%; X 0.25%; M 0.21%; D 0.20%; N 0.16%; Y 0.13%; F 0.11%; L 0.05%; H 0.03%; # 0.02%; W 0.02%
340	K	27.67%	E	18.36%	-9.31%	K 27.67%; N 27.49%; E 18.36%; D 8.20%; T 4.81%; R 4.73%; S 2.85%; Q 1.62%; A 1.44%; G 0.97%; I 0.75%; X 0.34%; H 0.29%; V 0.18%; Y 0.16%; M 0.10%; L 0.05%
644	S	13.35%	G	3.17%	-10.18%	T 22.10%; R 19.52%; N 15.72%; S 13.35%; E 7.04%; K 6.25%; D 6.22%; G 3.17%; Q 3.13%; A 0.92%; W 0.70%; M 0.51%; H 0.44%; X 0.25%; Y 0.25%; I 0.16%; L 0.16%; F 0.03%; C 0.02%; P 0.02%; V 0.02%
185	D	37.50%	N	26.90%	-10.60%	D 37.50%; N 26.90%; E 11.08%; G 9.42%; K 5.48%; S 4.34%; T 1.34%; R 0.70%; A 0.67%; H 0.54%; Q 0.41%; X 0.34%; I 0.29%; V 0.26%; P 0.23%; Y 0.13%; L 0.10%; F 0.08%; M 0.05%
188	N	12.32%	Q	0.98%	-11.34%	N 12.32%; S 4.38%; K 3.35%; D 2.39%; E 1.54%; Q 0.98%; T 0.90%; G 0.88%; R 0.54%; A 0.33%; V 0.28%; H 0.18%; I 0.16%; P 0.16%; X 0.10%; Y 0.10%; L 0.08%; M 0.05%; F 0.02%
135	N	32.35%	T	20.48%	-11.87%	N 32.35%; T 20.48%; K 12.27%; S 5.42%; V 5.04%; I 4.53%; R 4.34%; L 2.96%; G 2.21%; E 2.11%; D 1.65%; Q 1.10%; A 0.98%; P 0.83%; W 0.77%; X 0.69%; Y 0.61%; H 0.41%; C 0.29%; F 0.28%; M 0.23%
640	D	19.73%	Q	7.82%	-11.91%	N 22.84%; G 20.44%; D 19.73%; S 11.39%; Q 7.82%; E 6.92%; K 3.01%; R 1.69%; H 1.59%; Y 1.31%; T 1.13%; A 0.79%; X 0.64%; V 0.21%; L 0.20%; I 0.13%; F 0.10%; M 0.02%; P 0.02%
161	I	35.85%	M	19.22%	-16.63%	I 35.85%; V 22.91%; M 19.22%; T 11.04%; A 9.20%; S 0.67%; X 0.21%; F 0.20%; L 0.15%; Y 0.13%; N 0.07%; R 0.07%; K 0.03%; E 0.02%; G 0.02%; P 0.02%
336	T	35.00%	A	17.78%	-17.22%	T 35.00%; A 17.78%; E 10.57%; K 8.16%; S 7.89%; G 4.84%; D 3.14%; Q 2.39%; R 2.11%; V 1.95%; N 1.64%; I 1.18%; X 0.56%; L 0.46%; P 0.33%; H 0.23%; M 0.18%; Y 0.11%; W 0.05%; # 0.02%; F 0.02%
620	D	27.26%	S	9.83%	-17.43%	D 27.26%; E 23.54%; N 16.48%; S 9.83%; T 8.30%; K 4.52%; G 3.16%; A 2.26%; Q 1.85%; H 1.10%; R 0.49%; X 0.47%; P 0.21%; I 0.20%; V 0.10%; L 0.07%; W 0.05%; Y 0.05%; # 0.02%; C 0.02%
142	N	20.75%	D	2.45%	-18.30%	N 20.75%; T 19.00%; S 11.60%; A 3.62%; G 3.47%; D 2.45%; I 2.24%; V 1.90%; K 1.73%; E 0.98%; Y 0.97%; R 0.87%; X 0.77%; H 0.67%; Q 0.67%; L 0.59%; P 0.51%; C 0.46%; M 0.33%; F 0.29%; W 0.08%; # 0.02%; * 0.02%
141	T	21.35%	D	2.50%	-18.85%	N 22.87%; T 21.35%; S 11.47%; A 4.63%; V 3.52%; G 3.13%; D 2.50%; K 2.32%; I 2.23%; L 1.28%; R 1.18%; Y 1.03%; E 0.93%; Q 0.85%; X 0.64%; H 0.62%; C 0.57%; F 0.51%; P 0.46%; M 0.41%; W 0.23%
139	N	23.85%	I	2.78%	-21.07%	T 29.53%; N 23.85%; S 11.50%; A 5.78%; V 4.34%; G 3.42%; I 2.78%; D 2.57%; K 1.98%; L 1.03%; R 1.03%; C 0.92%; E 0.87%; Y 0.79%; H 0.65%; X 0.62%; F 0.52%; Q 0.38%; P 0.36%; M 0.21%; W 0.07%; # 0.02%
335	R	30.76%	K	9.16%	-21.60%	R 30.76%; G 22.73%; K 19.16%; E 13.71%; A 3.70%; S 3.37%; T 1.49%; I 1.16%; Q 0.88%; N 0.72%; L 0.43%; X 0.43%; V 0.41%; M 0.26%; F 0.16%; Y 0.15%; W 0.10%; D 0.08%; H 0.05%
343	Q	29.19%	G	4.74%	-24.45%	Q 29.19%; K 24.10%; E 13.01%; R 10.88%; G 4.74%; H 3.99%; N 3.57%; T 2.57%; Y 2.19%; S 1.70%; D 0.92%; I 0.75%; A 0.64%; L 0.61%; X 0.34%; V 0.33%; W 0.15%; F 0.13%; M 0.07%; P 0.05%; # 0.02%; * 0.02%
270	I	61.14%	V	36.50%	-24.64%	I 61.14%; V 36.50%; T 1.54%; M 0.20%; A 0.13%; L 0.13%; E 0.10%; X 0.10%; D 0.05%; P 0.05%; F 0.02%; K 0.02%; N 0.02%
363	P	34.38%	N	8.92%	-25.46%	P 34.38%; Q 21.99%; S 17.41%; N 8.92%; H 5.56%; K 4.84%; R 2.75%; A 1.13%; E 0.97%; T 0.92%; G 0.41%; X 0.25%; D 0.15%; L 0.13%; I 0.08%; M 0.02%; V 0.02%; Y 0.02%
344	Q	45.21%	K	17.93%	-27.28%	Q 45.21%; K 17.93%; R 15.89%; E 6.66%; N 3.71%; G 2.63%; L 2.59%; M 1.57%; W 1.24%; H 0.82%; D 0.51%; A 0.28%; S 0.26%; Y 0.23%; X 0.20%; T 0.07%; V 0.07%; I 0.05%; F 0.02%; P 0.02%
396	N	34.51%	I	4.71%	-29.80%	N 34.51%; T 9.28%; L 6.63%; S 6.20%; I 4.71%; Y 4.60%; D 4.52%; M 3.75%; K 3.32%; E 3.06%; P 2.91%; F 2.85%; G 2.80%; H 2.13%; R 1.88%; V 1.64%; W 1.60%; Q 0.93%; A 0.82%; X 0.46%; C 0.07%; * 0.03%
460	N	36.50%	S	6.32%	-30.18%	N 36.50%; T 7.98%; K 7.71%; S 6.32%; G 6.28%; I 5.89%; D 5.69%; E 4.30%; V 3.27%; A 2.47%; Q 2.27%; R 1.96%; H 1.67%; L 1.36%; P 1.24%; Y 0.83%; X 0.49%; M 0.39%; F 0.16%; C 0.08%; * 0.02%; W 0.02%
461	N	39.79%	T	9.51%	-30.28%	N 39.79%; G 10.19%; T 9.51%; D 8.38%; S 8.13%; E 6.18%; K 3.55%; I 1.83%; R 1.37%; H 1.26%; V 1.24%; Q 1.11%; A 1.08%; P 0.87%; X 0.47%; L 0.46%; M 0.33%; Y 0.28%; C 0.05%; F 0.05%; W 0.02%
337	K	34.73%	T	1.46%	-33.27%	K 34.73%; E 16.80%; Q 12.19%; D 9.75%; N 7.95%; A 7.67%; R 2.55%; G 1.49%; T 1.46%; S 1.42%; L 0.98%; H 0.67%; I 0.51%; X 0.43%; V 0.39%; M 0.08%; Y 0.02%
271	I	44.39%	M	8.84%	-35.55%	I 44.39%; V 41.20%; M 8.84%; K 1.80%; R 1.39%; T 0.98%; A 0.67%; Q 0.25%; E 0.16%; L 0.13%; X 0.08%; S 0.05%; D 0.02%; G 0.02%
240	K	36.91%	P	0.15%	-36.76%	K 36.91%; T 22.92%; N 18.26%; R 6.18%; H 5.51%; Q 3.17%; S 2.93%; E 0.95%; Y 0.69%; G 0.56%; L 0.47%; A 0.39%; X 0.34%; D 0.16%; I 0.16%; P 0.15%; M 0.10%; C 0.05%; V 0.03%; F 0.02%
154	I	42.21%	L	4.38%	-37.83%	I 42.21%; M 37.22%; V 14.01%; L 4.38%; R 0.65%; T 0.34%; X 0.31%; A 0.23%; Q 0.15%; E 0.11%; F 0.10%; K 0.10%; G 0.03%; N 0.03%; S 0.03%; W 0.03%; Y 0.03%
507	E	45.17%	G	3.89%	-41.28%	E 45.17%; Q 44.96%; G 3.89%; K 1.93%; D 1.69%; R 0.69%; A 0.52%; T 0.51%; N 0.20%; S 0.11%; H 0.10%; P 0.05%; L 0.03%; V 0.03%; X 0.03%; M 0.02%; W 0.02%
588	K	63.11%	R	21.53%	-41.58%	K 63.11%; R 21.53%; Q 11.08%; G 1.19%; S 0.56%; E 0.49%; H 0.44%; T 0.36%; A 0.26%; N 0.23%; M 0.20%; X 0.20%; V 0.10%; W 0.10%; Y 0.10%; I 0.03%; C 0.02%; L 0.02%
183	P	63.45%	Q	20.81%	-42.64%	P 63.45%; Q 20.81%; S 6.58%; K 3.14%; E 2.08%; A 1.00%; L 0.92%; R 0.54%; H 0.38%; N 0.29%; T 0.21%; G 0.20%; X 0.10%; D 0.07%; V 0.03%; # 0.02%; I 0.02%; Y 0.02%
333	I	65.12%	V	20.98%	-44.14%	I 65.12%; V 20.98%; L 13.53%; M 0.11%; X 0.08%; F 0.05%; T 0.05%; N 0.02%; P 0.02%; S 0.02%
351	E	63.60%	K	16.56%	-47.04%	E 63.60%; K 16.56%; G 3.83%; T 2.81%; D 2.47%; S 2.19%; Q 1.82%; N 1.70%; A 1.51%; R 1.21%; V 0.72%; I 0.36%; X 0.29%; P 0.28%; H 0.25%; L 0.16%; Y 0.07%; F 0.03%; M 0.03%; # 0.02%; C 0.02%
413	T	53.08%	S	2.05%	-51.03%	T 53.08%; N 25.88%; I 7.15%; V 2.55%; P 2.36%; S 2.05%; D 1.67%; K 0.79%; E 0.74%; L 0.59%; H 0.56%; Y 0.54%; X 0.43%; A 0.29%; F 0.26%; M 0.15%; R 0.15%; Q 0.08%; C 0.02%; G 0.02%; W 0.02%
283	T	67.67%	N	12.68%	-54.99%	T 67.67%; I 13.74%; N 12.68%; V 4.25%; S 1.03%; A 0.23%; P 0.15%; X 0.08%; D 0.03%; M 0.03%; # 0.02%; G 0.02%; K 0.02%; L 0.02%; Y 0.02%
629	M	71.14%	L	10.98%	-60.16%	M 71.14%; I 11.16%; L 10.98%; T 2.73%; V 1.51%; R 1.03%; Q 0.57%; K 0.34%; N 0.26%; A 0.15%; X 0.07%; # 0.02%; C 0.02%; W 0.02%
356	K	70.40%	N	2.34%	-68.06%	K 70.40%; T 14.25%; R 5.69%; N 2.34%; S 2.26%; A 1.69%; E 0.85%; G 0.82%; I 0.39%; Q 0.38%; X 0.25%; D 0.20%; P 0.10%; V 0.08%; M 0.07%; H 0.05%; L 0.03%; # 0.02%; F 0.02%; Y 0.02%
130	N	69.00%	Q	0.88%	-68.12%	N 69.00%; E 6.05%; D 5.64%; H 5.58%; K 4.74%; T 2.96%; S 1.85%; R 1.18%; I 0.90%; Q 0.88%; Y 0.38%; X 0.29%; L 0.21%; M 0.11%; # 0.07%; A 0.03%; F 0.03%; V 0.03%; C 0.02%; W 0.02%
92	N	75.18%	E	6.90%	-68.28%	N 75.18%; E 6.90%; D 6.64%; K 4.14%; Y 2.55%; S 1.26%; T 0.88%; P 0.79%; H 0.57%; Q 0.39%; R 0.21%; G 0.13%; X 0.13%; A 0.05%; M 0.05%; V 0.05%; I 0.03%; L 0.02%; W 0.02%
453	L	84.00%	I	14.92%	-69.08%	L 84.00%; I 14.92%; M 0.52%; V 0.23%; F 0.08%; X 0.07%; G 0.05%; P 0.03%; R 0.02%; S 0.02%; T 0.02%; Y 0.02%
348	K	81.14%	Q	11.71%	-69.43%	K 81.14%; Q 11.71%; E 3.98%; R 1.23%; T 0.52%; A 0.36%; L 0.26%; I 0.16%; X 0.15%; N 0.11%; S 0.11%; H 0.08%; V 0.07%; # 0.03%; F 0.02%; G 0.02%; M 0.02%
617	K	84.88%	R	13.40%	-71.48%	K 84.88%; R 13.40%; T 0.52%; S 0.21%; N 0.11%; X 0.11%; I 0.10%; E 0.08%; L 0.08%; G 0.07%; D 0.03%; V 0.03%; Y 0.03%; * 0.02%; H 0.02%; M 0.02%; Q 0.02%
618	S	77.08%	N	4.71%	-72.37%	S 77.08%; T 16.87%; N 4.71%; P 0.34%; D 0.16%; A 0.15%; X 0.11%; Y 0.11%; K 0.10%; E 0.07%; G 0.05%; L 0.05%; # 0.02%; F 0.02%; Q 0.02%; R 0.02%; V 0.02%
371	I	86.34%	V	11.68%	-74.66%	I 86.34%; V 11.68%; L 0.93%; T 0.64%; M 0.11%; X 0.10%; S 0.05%; N 0.03%; # 0.02%; D 0.02%; F 0.02%; Q 0.02%; R 0.02%
567	Q	85.24%	K	10.50%	-74.74%	Q 85.24%; K 10.50%; R 4.06%; X 0.07%; E 0.05%; L 0.05%; N 0.02%; P 0.02%
174	A	88.45%	S	10.75%	-77.70%	A 88.45%; S 10.75%; V 0.28%; T 0.20%; G 0.18%; D 0.03%; E 0.03%; K 0.03%; X 0.03%; P 0.02%
288	L	92.52%	F	7.28%	-85.24%	L 92.52%; F 7.28%; M 0.07%; X 0.03%; # 0.02%; P 0.02%; S 0.02%; V 0.02%; W 0.02%
430	V	90.92%	I	1.11%	-89.81%	V 90.92%; A 4.55%; T 2.83%; I 1.11%; L 0.20%; G 0.11%; P 0.11%; E 0.08%; * 0.03%; S 0.02%
285	I	97.32%	L	2.09%	-95.23%	I 97.32%; L 2.09%; M 0.33%; T 0.15%; V 0.07%; A 0.02%; X 0.02%
241	N	97.19%	S	0.39%	-96.80%	N 97.19%; D 0.98%; K 0.92%; S 0.39%; E 0.15%; H 0.07%; T 0.07%; X 0.05%; Y 0.05%; I 0.03%; Q 0.03%; # 0.02%; C 0.02%; G 0.02%; M 0.02%; R 0.02%

Supplementary Table 3. Amino acid conservation analysis of ConM and BG505 sequences used for generating Fig. 2c

Tier categorization

Tier Phenotyping GMT Window 1		Tier Phenotyping GMT Window 2	
**Tier 1A**	>1000	**Tier 1A**	>1000
**Tier 1B**	250-1000	**Tier 1B**	400-700
**Tier 2**	50-250	**Tier 2**	100-400
**Tier 3**	<50	**Tier 3**	<100

	**ConM**		**ConS**
	**Tier 1A**		**Tier 1B**
**Sample ID**	**ID$_{50}$**	**Sample ID**	**ID$_{50}$**
CHAVI-0406 pool	6111	CHAVI-0537 pool	1788
CHAVI-0060 pool	655	CHAVI-0468 pool	72
CHAVI-0642 pool	3909	CHAVI-0461 pool	196
CHAVI-0293 pool	3931	CHAVI-0383 pool	270
CHAVI-0598 pool	43740	CHAVI-0134 pool	4962
CHAVI-0585 pool	135	GMT	508
GMT	2671		

Antibody neutralization

		**ConM**		**ConS**
		**Tier 1A**		**Tier 1B**
**Antibody**	**Epitope**	**ID$_{50}$**	**Antibody**	**ID$_{50}$**
2219	V3	>25	2219	>25
2557	V3	23	2557	>25
3074	V3	13.2	3074	>25
3869	V3	23.6	3869	>25
447-52D	V3	>25	447-52D	>25
838-12D	V3	23	838-12D	>25
830A	V2	>25	830A	N.A.
654-30D	CD4bs	24.1	654-30D	>25
1008-30D	CD4bs	>25	1008-30D	>25
1570D	CD4bs	14	1570D	>25
729-30D	CD4bs	19.5	729-30D	24.4
F105	CD4bs	N.A.	F105	>25
181D	gp41(cluster I)	16.7	181D	N.A.
246D	gp41(cluster I)	15.9	246D	N.A.
240D	gp41(cluster I)	>25	240D	N.A.
50-69D	gp41(cluster I)	6.2	50-69D	N.A.
sCD4	CD4bs	21.2	sCD4	18.6
VRC01	CD4bs	0.19	VRC01	1.44
3BNC117	CD4bs	<0.01	3BNC117	0.25
CH31	CD4bs	0.53	CH31	1.2
PG9	V2-glycan	0.03	PG9	0.03
PG16	V2-glycan	0.005	PG16	0.02
CH01	V2-glycan	14.8	CH01	12.2
PGDM1400	V2-glycan	N.A.	PGDM1400	0.05
PGT128	V3-glycan	<0.002	PGT128	0.01
PGT121	V3-glycan	0.003	PGT121	0.02
10-1074	V3-glycan	<0.01	10-1074	<0.01
PGT151	gp120/gp41	<0.002	PGT151	<0.002
VRC34.01	gp120/gp41	N.A.	VRC34.01	0.2
2G12	glycan	>25	2G12	3.33
DH511.2.K3_4a	MPER	N.A.	DH511.2.K3_4a	0.23
10E8	MPER	N.A.	10E8	0.65
2F5	MPER	N.A.	2F5	1.23
4E10	MPER	1.69	4E10	2.17
HIVIG-C		4.83	HIVIG-C	29
CH01-31		N.A.	CH01-31	0.97

Supplementary Table 4. Characterization of the ConM and ConS viruses. Top: tier categorization of the ConM and ConS viruses using a panel of reference sera pool. Bottom: analysis of the sensitivity of ConM and ConS to a panel of bNAbs and non-NAbs. The TZM-bl cell assays were performed at DUMC. GMT: geometric mean titer. N.A: not tested.

Week 22	Virus	MLV	MLV	ConM	ConM	ConM	ConM	SF162	SF162	MW965.26	AMC008	ZM197M	ConS	ConS	ConS
		Control		Autologous				Heterologous Tier 1							
	Tier			1A	1A	1A	1A	1A	1A	1A	1B	1B	1B	1B	1B
	Clade							B	B	C	B	C			
Immunogen	Lab	AMC	DUMC	AMC	DUMC	OSR	ISCIII	AMC	ISCIII	DUMC	AMC	AMC	AMC	DUMC	OSR
ConM SOSIP.v7 (ISCOMATRIX™)	1839	<20	<20	56944	46649	58493	49939	29	<20	449	<20	<20	231	109	175
	1840	<20	<20	146656	143538	72497	88150	26	<20	218	<20	<20	308	82	127
	1841	<20	<20	80585	64681	48667	91919	<20	<20	116	<20	<20	123	54	44
	1842	<20	<20	16010	16106	10482	24314	24	62	114	<20	<20	71	40	216
	1843	<20	<20	15008	26484	13017	43449	27	26	76	<20	<20	126	57	43
ConM SOSIP.v7-ferritin (ISCOMATRIX™)	1809	<20	<20	31880	36017	28178	21227	52	27	21599	<20	<20	2163	1149	797
	1810	<20	<20	25953	56137	16014	8007	61	28	3732	<20	<20	391	290	696
	1811	<20	<20	44432	70273	26597	10609	583	72	9982	<20	<20	3321	1344	1485
	1812	<20	<20	203036	164177	152493	43901	1437	405	3486	<20	<20	6199	3090	2639
	1813	<20	<20	449906	354473	245476	125261	274	58	29493	<20	<20	3993	3141	1399
ConM SOSIP.v7 (SE)	1.1	<20		5110		2107		<20					423		116
	1.2	<20		6757		3012		<20					448		83
	1.3	<20		16917		3893		<20					264		33
	1.4	<20		82485		34782		<20					327		64
	1.5	<20		809		204		<20					65		20
	1.6	<20		6162		6944		<20					64		166
ConM SOSIP.v7-ferritin (SE)	4.1	23		79605		52368		488					3548		757
	4.2	<20		52461		16086		192					1877		496
	4.3	<20		34131		8033		691					1771		1211
	4.4	<20		86348		16255		8650					1072		126
	4.5	<20		117823		23534		178					880		382
	4.6	<20		82326		25108		1231					2306		782
	Rabbit ID														

Week 22	Virus	BG505	246-F3_C10_2 [a]	CNE55 [a]	REJO	SHIVp3	SHIVp3	TRO.11	WITO	BJOX002000.03.2 [a]	CH119.10 [a]	25710-2.43	Ce1176_A3	Ce703010217_B6 [a]	TV1.21	TV1.21	TV1.29	X1632-S2-B10 [a]
		Heterologous Tier 2																
	Tier	2	2	2	2	2	2	2	2	2	2	2	2	2	2	2	2	2
	Clade	A	AC	CRF01_AE	B	B	B	B	B	CRF07_BC	CRF07_BC	C	C	C	C	C	C	G
Immunogen	Lab	AMC	DUMC	DUMC	AMC	AMC	OSR	DUMC	AMC	DUMC	DUMC	DUMC	DUMC	DUMC	AMC	DUMC	OSR	DUMC
ConM SOSIP.v7 (ISCOMATRIX™)	1839	<20	<20	<20	<20	<20		<20	<20	<20	<20	<20	<20	<20		<20		<20
	1840	<20	<20	<20	34	37		<20	<20	<20	<20	<20	<20	<20		<20		<20
	1841	<20	<20	<20	20	<20		<20	<20	<20	<20	<20	<20	<20		<20		<20
	1842	<20	<20	<20	40	<20		<20	<20	<20	<20	<20	<20	<20		<20		<20
	1843	<20	<20	<20	<20	<20		<20	<20	<20	<20	<20	<20	<20		<20		<20
ConM SOSIP.v7-ferritin (ISCOMATRIX™)	1809	<20	<20	<20	<20	<20		<20	<20	<20	<20	<20	<20	<20		207		<20
	1810	<20	34	<20	<20	<20		<20	<20	26	23	<20	<20	<20		107		<20
	1811	<20	<20	<20	26	62		<20	<20	<20	<20	<20	<20	<20		158		<20
	1812	<20	<20	<20	<20	<20		<20	<20	<20	<20	<20	<20	<20		65		<20
	1813	<20	78	<20	<20	<20		<20	<20	73	<20	<20	<20	<20		410		<20
ConM SOSIP.v7 (SE)	1.1						<20								23		<20	
	1.2						<20								33		<20	
	1.3						<20								<20		<20	
	1.4						<20								24		<20	
	1.5						<20								24		<20	
	1.6						<20								27		<20	
ConM SOSIP.v7-ferritin (SE)	4.1						<20								127		<20	
	4.2						<20								<20		<20	
	4.3						<20								422		<20	
	4.4						<20								169		<20	
	4.5						<20								60		<20	
	4.6						<20								123		<20	

Supplementary Table 5. Midpoint neutralization titers for sera from rabbits immunized with ConM SOSIP.v7 or ConM SOSIP.v7-ferritin. Sera (from week 22, unless otherwise noted) were tested against a panel of Env-pseudotyped viruses in a standard TZM-bl assay. Upper part: neutralization titers for sera from rabbits immunized with ConM SOSIP.v7 or ConM SOSIP.v7-ferritin receiving ISCOMATRIX™ adjuvant. Immunizations were carried out by Covance (Denver, USA). The neutralization assays were performed at AMC, DUMC, OSR and ISCIII.

Lower part: neutralization titers for week 22 sera from rabbits immunized with ConM SOSIP.v7 or ConM SOSIP.v7-ferritin formulated with Squalene Emulsion (SE) adjuvant. Immunizations were carried out by NFCSO-DVMP (Gödölló, Hungary). The TZM-bl cell assays were performed at AMC and OSR.

The boxes are colored according to their magnitude. ID50 <20 in white; ID50 20-40 in grey; ID50 40-100 in yellow; ID50 100-1000 in orange; ID50 1000-10000 in red; ID50 10000-100000 in pink; ID50 >100000 in purple; [a]week 38 sera dilutions.

			Autologous		
Week 22	Virus	MLV	ConM	ConM	ConM
	Tier		1A	1A	1A
Immunogen	Dump-in Protein			V3 Peptide	ConM D368R
ConM SOSIP.v7	1839	<20	>4860	>4860	46
	1840	31	>4860	>4860	53
	1841	<20	>4860	>4860	55
	1842	<20	>4860	>4860	26
	1843	<20	>4860	>4860	<20
ConM SOSIP.v7-ferritin	1809	<20	>4860	>4860	49
	1810	<20	>4860	>4860	45
	1811	<20	>4860	>4860	45
	1812	<20	>4860	>4860	38
	1813	<20	>4860	>4860	135
	Rabbit ID				

Supplementary Table 6. Neutralization depletion experiment with ConM virus. Midpoint neutralization titers for sera from rabbits against ConM virus with or without cyclic ConM V3 peptide (which is identical to that of BG505) and ConM SOSIP.v7 D368R trimer as competitors.

Week 22	Virus	ConS [a]											TV1.21					TV1.21
	Mutant		Y173A	T236K	N295A	N301A	G324A	N332A	V518L	T607S	T607K	W680R K683Q		N160A	N332A	K169E	N276A	
			V1V2 glycan	gp120-gp41	2G12 sens	V3 glycan	V3 glycan	V3 glycan	fusion peptide	gp120-gp41	gp120-gp41	MPER		V1V2 glycan	V3 glycan	V1V2 glycan	CD4BS	
Immunogen	Clade												C	C	C	C	C	C
ConM SOSIP.v7	1839	100	25	120	110	<20	<20	118	161	234	385	81						<20
	1840	79	<20	85	81	<20	<20	109	88	133	146	67						<20
	1841	52	<20	61	43	<20	<20	72	51	62	52	38						<20
	1842	57	<20	69	56	<20	<20	55	73	79	109	42						<20
	1843	57	28	65	62	<20	<20	82	64	79	83	46						<20
ConM SOSIP.v7-ferritin	1809	519	87	489	422	266	26	697	461	347	553	403	121	312	782	<20	146	207
	1810	209	108	209	189	97	42	238	201	172	250	176	81	148	317	<20	83	107
	1811	624	306	566	390	244	54	878	519	501	678	466	142	332	973	<20	179	158
	1812	1333	169	1284	1012	268	44	1990	989	1265	1675	1065	48	102	222	<20	80	65
	1813	1689	922	1989	2102	1336	106	3148	1230	1566	1652	1616	290	588	1210	<20	333	410
ConM SOSIP.v7 (fold change)	1839		4.0	0.8	0.9	5.0	5.0	0.8	0.6	0.4	0.3	1.2						
	1840		4.0	0.9	1.0	4.0	4.0	0.7	0.9	0.6	0.5	1.2						
	1841		2.6	0.9	1.2	2.6	2.6	0.7	1.0	0.8	1.0	1.4						
	1842		2.9	0.8	1.0	2.9	2.9	1.0	0.8	0.7	0.5	1.4						
	1843		2.0	0.9	0.9	2.9	2.9	0.7	0.9	0.7	0.7	1.2						
ConM SOSIP.v7-ferritin (fold change)	1809		6.0	1.1	1.2	2.0	20.0	0.7	1.1	1.5	0.9	1.3		0.4	0.2	6.1	0.8	
	1810		1.9	1.0	1.1	2.2	5.0	0.9	1.0	1.2	0.8	1.2		0.5	0.3	4.1	1.0	
	1811		2.0	1.1	1.6	2.6	11.6	0.7	1.2	1.2	0.9	1.3		0.4	0.1	7.1	0.8	
	1812		7.9	1.0	1.3	5.0	30.3	0.7	1.3	1.1	0.8	1.3		0.5	0.2	2.4	0.6	
	1813		1.8	0.8	0.8	1.3	15.9	0.5	1.4	1.1	1.0	1.0		0.5	0.2	14.5	0.9	
	Rabbit ID																	

Immunogen	Rabbit ID	MW965.26			SF162.LS			TV1.21					
								N160A			N332A		
depletion peptide		-	scramble	ConM V3	-	scramble	ConM V3	-	scramble	ConM V3	-	scramble	ConM V3
	Clade	C	C	C	B	B	B	C	C	C	C	C	C
ConM SOSIP.v7	1839	861	849	359	194	210	138	<20	<20	<20	<20	<20	<20
	1840	883	1051	441	450	428	327	162	153	115	287	171	170
	1841	372	472	170	50	62	42	102	107	79	151	143	112
	1842	330	304	212	180	172	129	87	75	93	114	123	103
	1843	276	326	200	236	206	114	96	79	91	123	121	122
ConM SOSIP.v7-ferritin	1809	18574	13341	3326	801	601	181	458	422	187	1246	715	281
	1810	3973	2671	813	256	209	201	214	175	138	412	341	186
	1811	11760	6534	1647	1889	1062	509	394	304	194	891	902	286
	1812	3850	2921	1452	2367	3312	730	211	154	129	326	325	193
	1813	23015	22572	3181	625	462	266	1109	719	338	2931	1866	635
ConM SOSIP.v7 (fold change)	1839		1.0	2.4		0.9	1.4						
	1840		0.8	2.0		1.1	1.4		1.1	1.4		1.7	1.7
	1841		0.8	2.2		0.8	1.2		1.0	1.3		1.1	1.3
	1842		1.1	1.6		1.0	1.4		1.2	0.9		0.9	1.1
	1843		0.8	1.4		1.1	2.1		1.2	1.1		1.0	1.0
ConM SOSIP.v7-ferritin (fold change)	1809		1.4	5.6		1.3	4.4		1.1	2.4		1.7	4.4
	1810		1.5	4.9		1.2	1.3		1.2	1.6		1.2	2.2
	1811		1.8	7.1		1.8	3.7		1.3	2.0		1.0	3.1
	1812		1.3	2.7		0.7	3.2		1.4	1.6		1.0	1.7
	1813		1.0	7.2		1.4	2.3		1.5	3.3		1.6	4.6

Supplementary Table 7. Epitope mapping of sera from ConM SOSIP.v7 immunized rabbits for ConS, TV1.21, MW965.26 and SF162.LS viruses. Left: midpoint neutralization titers for the sera against a panel of ConS and TV1.21 mutant viruses. Right: midpoint titers of MW965.26, SF162.LS and two TV1.21 mutants (N160A and N332A) after depleting the sera using scrambled or ConM V3 peptide. The TZM-bl cell assay was performed at DUMC. The lower part depicts the fold changes in ID50 titers compared to WT virus or non-depleted serum (ID50 virus/ID50 mutant virus). Fold change <0.25 in grey; 0.25-2.5 in white; 2.5-10 in light blue; >10 in dark blue. [a]week 38 sera dilutions.

		Control		Autologous				Heterologous Tier 1			
Week 22	Virus	MLV	MLV	ConM	ConM	AMC008	AMC011	SF162	MW965.26	ConS	ConS
	Tier			1A	1A	1B	2	1A	1A	1B	1B
	Clade					B	B	B	C		
Immunogen	Lab	AMC	DUMC	AMC	DUMC	AMC	AMC	AMC	DUMC	AMC	DUMC
SOSIP-ferritin cocktail	r05048	<20	23	1416	890	<20	<20	152	403	<20	117
	r10078	<20	<20	1750	801	<20	<20	286	490	<20	100
	r11043	<20	<20	>4860	2417	<20	<20	720	955	58	129
	r08003	<20	<20	>4860	5227	<20	<20	3124	3369	140	210
	r09093	<20	<20	>4860	1377	<20	<20	1248	801	103	148
	r10064	<20	<20	1923	2383	<20	<20	955	677	23	74
negative controls	r09038	<20	23	<20	<20	<20	<20	<20	<20	<20	<20
	r10045	<20	27	<20	<20	<20	<20	<20	<20	<20	<20
	Macaque ID										

		Heterologous Tier 2													
Week 22	Virus	398_F1_F6_20	246-F3_C10_2	CNE55	REJO	SHIVp3	TRO.11	X2278-C2-B6	BJOX002000.03.2	CH119.10	25710-2.43	Ce1176_A3	Ce703010217_B6	TV1.21	X1632-S2-B10
	Tier	2	2	2	2	2	2	2	2	2	2	2	2	2	2
	Clade	A	AC	CRF01_AE	B	B	B	B	CRF07_BC	CRF07_BC	C	C	C	C	G
Immunogen	Lab	DUMC	DUMC	DUMC	AMC	AMC	DUMC	DUMC	DUMC	DUMC	DUMC	DUMC	DUMC	DUMC	DUMC
SOSIP-ferritin cocktail	r05048	26	<20	<20	<20	<20	<20	<20	<20	<20	<20	<20	<20	<20	<20
	r10078	22	<20	<20	<20	<20	<20	<20	<20	<20	<20	<20	<20	<20	<20
	r11043	<20	<20	<20	<20	<20	<20	<20	<20	<20	<20	<20	<20	<20	<20
	r08003	<20	<20	<20	<20	<20	<20	<20	<20	<20	<20	<20	<20	<20	<20
	r09093	<20	<20	<20	<20	<20	<20	<20	<20	<20	<20	<20	<20	<20	<20
	r10064	<20	<20	<20	<20	<20	<20	<20	<20	<20	<20	<20	<20	<20	<20
negative controls	r09038				<20	<20									
	r10045				<20	<20									
	Macaque ID														

Supplementary Table 8. Neutralization by sera from macaques immunized with a trivalent cocktail of SOSIP-ferritin nanoparticles.
Midpoint neutralization titers were determined for a panel of Env-pseudotyped viruses in the TZM-bl cell assay (performed at AMC and DUMC).

Week 22 plasma	Virus	MLV	ConS										TV1.21					MW965.26			SF162.LS		
	Mutant			Y173A	T236K	N295A	N301A	G324A	N332A	V518L	T607S	W680R K683Q		N160A	N332A	K169E	N276A						
				V1V2 glycan	gp120-gp41	2G12 sens	V3 glycan	V3 glycan	V3 glycan	fusion peptide	gp120-gp41	MPER		V1V2 glycan	V3 glycan	V1V2 glycan	CD4BS	-	scramble	ConM V3	-	scramble	ConM V3 depletion peptide
Immunogen	Clade	-											C	C	C	C	C	C	C	C	B	B	B
SOSIP-ferritin cocktail	r05048	70	458	297	294	203	172	468	127	158	143	343	48	56	79	<30	73	629	476	345	543	518	223
	r10078	57	459	227	249	478	363	417	285	442	140	529	60	47	78	<30	68	851	585	500	868	1059	367
	r11043	43	324	193	281	233	195	241	241	275	131	301	30	55	125	<30	74	1763	1074	397	1159	1116	574
	r08003	65	503	307	421	489	460	568	277	490	275	675	148	148	748	34	208	3915	4219	1541	5018	4548	2601
	r09093	45	287	240	204	234	168	325	132	206	150	295	61	58	110	<30	74	968	1064	569	1317	1184	657
	r10064	39	266	175	179	193	195	295	96	202	96	385	67	56	88	<30	75	849	752	461	1045	1116	430
negative controls	r09038	38	94	78	112	45	45	140	49	72	32	87	<20	<30	36	<30	40	53	58	48	52	40	34
	r10045	46	146	78	157	35	78	177	30	106	34	137	<20	<30	36	<30	40	64	68	54	69	59	110
SOSIP-ferritin cocktail (fold change)	r05048			1.5	1.6	2.3	2.7	1.0	3.6	2.9	3.2	1.3							1.3	1.8		1.0	2.4
	r10078			2.0	1.8	1.0	1.3	1.1	1.6	1.0	3.3	0.9							1.5	1.7		0.8	2.4
	r11043			1.7	1.2	1.4	1.7	1.3	1.3	1.2	2.5	1.1							1.6	4.4		1.0	2.0
	r08003			1.6	1.2	1.0	1.1	0.9	1.8	1.0	1.8	0.7							0.9	2.5		1.1	1.9
	r09093			1.2	1.4	1.2	1.7	0.9	2.2	1.4	1.9	1.0							0.9	1.7		1.0	2.0
	r10064			1.5	1.5	1.4	1.4	0.9	2.8	1.3	2.8	0.7							1.5	1.8		0.8	2.4
negative controls (fold change)	r09038			1.2	0.8	2.1	2.1	0.7	1.9	1.3	2.9	1.1							1.6	1.1		1.0	1.5
	r10045			1.9	0.9	4.2	1.9	0.8	4.9	1.4	4.3	1.1							0.9	1.2		1.1	0.6
	Macaque ID																						

Supplementary Table 9. Mapping of the neutralizing epitopes on ConS, TV1.21, MW965.26 and SF162.LS for immunized macaques. Midpoint neutralization titers of plasma from macaques immunized with a trivalent cocktail of SOSIP-ferritin against a panel of heterologous ConS and TV1.21 mutant viruses (left) and MW965.26 and SF162.LS depleting the sera using scrambled or ConM V3 peptide (right). The TZM-bl cell assays were performed at DUMC. Note the relatively high MLV neutralization titers.
Lower part: fold change in ID50 titers (ID50 virus/ID50 mutant virus). Fold change <0.25 in grey; 0.25-2.5 in white; 2.5-10 in light blue; >10 in dark blue.

Supplementary References

1. de Taeye, S. W. *et al.* Immunogenicity of stabilized HIV-1 envelope trimers with reduced exposure of non-neutralizing epitopes. *Cell* **163,** 1702–15 (2015).
2. Torrents de la Peña, A. *et al.* Improving the immunogenicity of native-like HIV-1 envelope trimers by hyperstabilization. *Cell Rep* **20,** 1805–1817 (2017).
3. Guenaga, J. *et al.* Structure-guided redesign increases the propensity of HIV Env to generate highly stable soluble trimers. *J. Virol.* **90,** 2806–17 (2015).
4. Behrens, A.-J. *et al.* Composition and antigenic effects of individual glycan sites of a trimeric HIV-1 envelope glycoprotein. *Cell Rep* **14,** 2695–706 (2016).
5. Stewart-Jones, G. B. E. *et al.* Trimeric HIV-1-Env structures define glycan shields from clades A, B, and G. *Cell* **165,** 813–26 (2016).
