# Supplementary material for: Structure and immunogenicity of a stabilized HIV-1 envelope trimer based on a group-M consensus sequence
Source: Nat Commun. 2019 May 29;10:2355. doi: 10.1038/s41467-019-10262-5 (PMC6541627; doi:10.1038/s41467-019-10262-5)
Supplement: Supplementary file 3 — Reporting Summary [file 41467_2019_10262_MOESM3_ESM.pdf]

nature research

Corresponding author(s): Rogier W. Sanders
Ian A. WIlson
Byung Woo Han

Reporting Summary

Nature Research wishes to improve the reproducibility of the work that we publish. This form provides structure for consistency and transparency in reporting. For further information on Nature Research policies, see Authors & Referees and the Editorial Policy Checklist.

Statistical parameters

When statistical analyses are reported, confirm that the following items are present in the relevant location (e.g. figure legend, table legend, main text, or Methods section).

n/a	Confirmed	
☐	☒	The exact sample size (n) for each experimental group/condition, given as a discrete number and unit of measurement
☐	☒	An indication of whether measurements were taken from distinct samples or whether the same sample was measured repeatedly
☐	☒	The statistical test(s) used AND whether they are one- or two-sided<br>*Only common tests should be described solely by name; describe more complex techniques in the Methods section.*
☒	☐	A description of all covariates tested
☐	☒	A description of any assumptions or corrections, such as tests of normality and adjustment for multiple comparisons
☐	☒	A full description of the statistics including central tendency (e.g. means) or other basic estimates (e.g. regression coefficient) AND variation (e.g. standard deviation) or associated estimates of uncertainty (e.g. confidence intervals)
☐	☒	For null hypothesis testing, the test statistic (e.g. F, t, r) with confidence intervals, effect sizes, degrees of freedom and P value noted<br>*Give P values as exact values whenever suitable.*
☒	☐	For Bayesian analysis, information on the choice of priors and Markov chain Monte Carlo settings
☒	☐	For hierarchical and complex designs, identification of the appropriate level for tests and full reporting of outcomes
☒	☐	Estimates of effect sizes (e.g. Cohen's d, Pearson's r), indicating how they were calculated
☐	☒	Clearly defined error bars<br>*State explicitly what error bars represent (e.g. SD, SE, CI)*

Our web collection on statistics for biologists may be useful.

Software and code

Policy information about availability of computer code

Data collection	Microsoft Excel for database management.<br>Crystallographic software is described in the methods section
Data analysis	Graphpad Prism 7.0

For manuscripts utilizing custom algorithms or software that are central to the research but not yet described in published literature, software must be made available to editors/reviewers upon request. We strongly encourage code deposition in a community repository (e.g. GitHub). See the Nature Research guidelines for submitting code & software for further information.

Data

Policy information about availability of data

All manuscripts must include a data availability statement. This statement should provide the following information, where applicable:

- Accession codes, unique identifiers, or web links for publicly available datasets
- A list of figures that have associated raw data
- A description of any restrictions on data availability

The data that support the findings of this study are available from the corresponding author upon reasonable request.
The coordinates and structure factors for the ConM SOSIP.v7 structure in complex with PGT124 and 35O22 have been deposited in the Protein Data Bank (PDB code

6IEQ).

Field-specific reporting

Please select the best fit for your research. If you are not sure, read the appropriate sections before making your selection.

- [x] Life sciences
- [ ] Behavioural & social sciences
- [ ] Ecological, evolutionary & environmental sciences

For a reference copy of the document with all sections, see nature.com/authors/policies/ReportingSummary-flat.pdf

Life sciences study design

All studies must disclose on these points even when the disclosure is negative.

Sample size	No sample-size calculations were performed.
Data exclusions	No data was excluded from analyses
Replication	Experiments were repeated and/or performed in duplicate.
Randomization	Allocation was random
Blinding	Blinding was not relevant for our studies

Reporting for specific materials, systems and methods

Materials & experimental systems

n/a	Involved in the study	
[ ]	[x]	Unique biological materials
[ ]	[x]	Antibodies
[ ]	[x]	Eukaryotic cell lines
[x]	[ ]	Palaeontology
[ ]	[x]	Animals and other organisms
[x]	[ ]	Human research participants

Methods

n/a	Involved in the study	
[x]	[ ]	ChIP-seq
[x]	[ ]	Flow cytometry
[x]	[ ]	MRI-based neuroimaging

Unique biological materials

Policy information about availability of materials

Obtaining unique materials	Unique materials are available upon request.

Antibodies

Antibodies used	All antibodies used were provided by Michel Nussenzweig, Hermann Katinger, Mark Connors, James Robinson, Dennis Burton, John Mascola, Peter Kwong and William Olson directly or through the NIH AIDS Research and Reference Reagent Program.
Validation	N.A.

Eukaryotic cell lines

Policy information about cell lines

Cell line source(s)	HEK293 cell lines were obtained from Thermo Fisher or ATCC as described in the methods.<br>B cell lines were provided by Takayuki Ota and David Nemazee
Authentication	None of the cell lines were validated
Mycoplasma contamination	All cell lines were tested negative for mycoplasma contamination

Commonly misidentified lines (See ICLAC register)	N.A.

Animals and other organisms

Policy information about studies involving animals; ARRIVE guidelines recommended for reporting animal research

Laboratory animals	New Zealand White rabbits, female, 2.5-3kg<br>Macaques, Macaca mulatta, female, 5-6 kg
Wild animals	N.A.
Field-collected samples	N.A.
